# HSP70 is required for the proper assembly of pericentriolar material and function of mitotic centrosomes

**DOI:** 10.1186/s13008-019-0047-7

**Published:** 2019-05-10

**Authors:** Chieh-Ting Fang, Hsiao-Hui Kuo, Shao-Chun Hsu, Ling-Huei Yih

**Affiliations:** 1 Department of Life Science, National Taiwan University, Taipei, Taiwan; 2 Institute of Cellular and Organismic Biology, Academia Sinica, Taipei, 115 Taiwan

**Keywords:** HSP70, Mitotic centrosome, Pericentriolar material, Spindle pole

## Abstract

**Background:**

At the onset of mitosis, the centrosome expands and matures, acquiring enhanced activities for microtubule nucleation and assembly of a functional bipolar mitotic spindle. However, the mechanisms that regulate centrosome expansion and maturation are largely unknown. Previously, we demonstrated in an immortalized human cell line CGL2 and cancer cell line HeLa that the inducible form of heat shock protein 70 (HSP70) accumulates at the mitotic centrosome and is required for centrosome maturation and bipolar spindle assembly.

**Results:**

In this study, we further show that HSP70 accumulated at the spindle pole in a PLK1-dependent manner. HSP70 colocalized with pericentrin (PCNT), CEP215 and γ-tubulin at the spindle pole and was required for the 3D assembly of these three proteins, which supports mitotic centrosome function. Loss of HSP70 disrupted mitotic centrosome structure, reduced pericentriolar material recruitment and induced fragmentation of spindle poles. In addition, HSP70 was necessary for the interaction between PCNT and CEP215 and also facilitated PLK1 accumulation and function at the spindle pole. Furthermore, we found that HSP70 chaperone activity is required for PCNT accumulation at the mitotic centrosome and assembly of mitotic spindles.

**Conclusion:**

Our current results demonstrate that HSP70 is required for the accurate assembly of the pericentriolar material and proper functioning of mitotic centrosomes.

## Background

The centrosome is a structurally complex and functionally diverse organelle that regulates various cellular processes. It consists of a pair of barrel-shaped structures called centrioles and a surrounding protein complex, which is referred to as the pericentriolar material (PCM). The PCM contains hundreds of proteins, including signaling molecules, cell cycle regulators and crucial microtubule (MT)-nucleating factors. The combined function of these proteins makes the centrosome the major MT-organizing center (MTOC), which coordinates all MT-related functions, including cell shape, cell polarity, mobility, intracellular trafficking and cell division [1]. The centrosome is duplicated during S phase along with DNA replication, yielding two centrosomes that nucleate extensive MT arrays and form the two poles of the mitotic spindle [2]. Importantly, from late S phase to mitotic onset, the two fully duplicated centrosomes exhibit enhanced recruitment of PCM components that are essential for MT nucleation, a process termed centrosome maturation. In this process, PCM components accumulate at the existing centrosome and expand into a greatly enlarged and intermingled matrix structure to enhance MT nucleation capacity and promote the assembly of a functional bipolar spindle [3–5]. Inaccurate accumulation of PCM components at the centrosome results in a functionally compromised mitotic centrosome with multipolar or disorganized spindles that may further promote mitotic arrest, cell death and/or aneuploidy [6, 7].

Previous studies using subdiffraction resolution microscopy and fluorescence recovery after photobleaching have revealed that PCM components adopt either a concentric toroidal or an extending fiber-like organization to form a thin layer of PCM at the interphase centrosome; these structures then undergo extensive rearrangement, continuous replenishment, local trafficking, massive accumulation and expansion, and occupy distinct subdomains within the dramatically enlarged mitotic PCM matrix [3, 4, 8–13]. While the mechanisms that precisely regulate the behaviors of PCM components are not clearly understood, several major contributors have been identified. For example, Polo like kinase 1 (PLK1) is a master mitotic kinase that is known to phosphorylate multiple PCM substrates and drive several steps of centrosome maturation [14–16]. Additionally, pericentrin (PCNT) and CEP215 and their respective homologues in various model organism are putative scaffold proteins that are thought to physically interact at the maturing centrosome, providing a proper platform to recruit other PCMs [15, 17–19]. In the PCM matrix, PCNT phosphorylation by PLK1 was shown to increase recruitment of MT nucleating factors, such as NEDD1 and γ-tubulin, whose accumulation is also PLK1-dependent [15, 17]. Notably, PLK1 activity persists throughout mitosis to ensure continuous replenishment of PCM components, including PLK1 itself, suggesting a highly dynamic and interdependent orchestration of PCM components at the functional mitotic centrosome [20, 21]. Despite these major advances in understanding centrosome maturation, the highly organized regulatory mechanisms governing the concentration and exchange of these and other proteins at the mitotic centrosome are still largely unknown. Improper accumulation of γ-tubulin has been implicated in abnormal spindle formation and human malignancies [22, 23]. Moreover, blocking PLK1 activity in the mitotic cells with already matured centrosomes reduces PCNT and γ-tubulin accumulation [21], while mutant PLK1 with an altered exchange rate at the mitotic centrosome induces mitotic arrest [20]. Mutant PCNT that cannot be phosphorylated by PLK1 fails to recruit multiple PCM components, including PLK1 itself [15], while mutations that abolish interaction between PCNT and CEP215 disrupt the recruitment of themselves and γ-tubulin, inducing the formation of defective mitotic centrosomes and spindles [17]. Together, these data imply that delicate proteostatic control of PCM components is important for determining the proper function of a fully matured mitotic centrosome.

Molecular chaperones, such as heat shock proteins (HSPs), are major orchestrators of the cellular proteostatic mechanisms that control protein quality through folding, oligomerization, trafficking, disaggregation, and proteasomal or autophagic degradation. Among the wide variety of HSPs, HSP70 family members form a central hub of the chaperone networks that cooperatively utilize various chaperones and co-chaperones to regulate all aspects of cellular proteostasis, especially protein quality control in certain organelles [24, 25]. HSP70 members in *S. cerevisiae* were proposed to control the oligomeric state of Cin8 motor protein to regulate spindle length [26]. In mammalian cells, HSP70 mainly exists in constitutive form (HSC70) and inducible form. The inducible HSP70 (thereafter HSP70) is phosphorylated by NEK6, which targets HSP70 to the mitotic spindle where it maintains kinetochore-MT stability and supports centrosome clustering [27, 28]. Additionally, HSP70 was also shown to protect centrosome and spindle integrity when overexpressed, likely by preventing proteasomal degradation of centrosomal proteins in the heat-shocked cells [29, 30]. Thus, the importance of HSP70 in the mitotic centrosome is well established, and some evidence suggests that it may exert chaperone activities on centrosome components to regulate centrosome maturation.

We have previously demonstrated that HSP70 (encoded by genes *HSPA1A* and *HSPA1B*) localizes to the mitotic spindle poles and is required for maintenance of centrosome integrity, MT nucleation, mitotic spindle assembly, mitotic progression, and cell viability [31]. In this study, we further employed subdiffraction resolution microscopy to examine the roles of HSP70 in the structure and function of the mitotic centrosome. We found that HSP70 cooperates with PLK1, PCNT, and CEP215 to support their accumulation and proper 3D assembly, and this action appears to be required for complete maturation of a fully functional mitotic centrosome.

## Results

### HSP70 accumulates at the spindle pole in a PLK1-dependent manner and colocalizes with PCNT, CEP215 and γ-tubulin

**Fig. 1:**
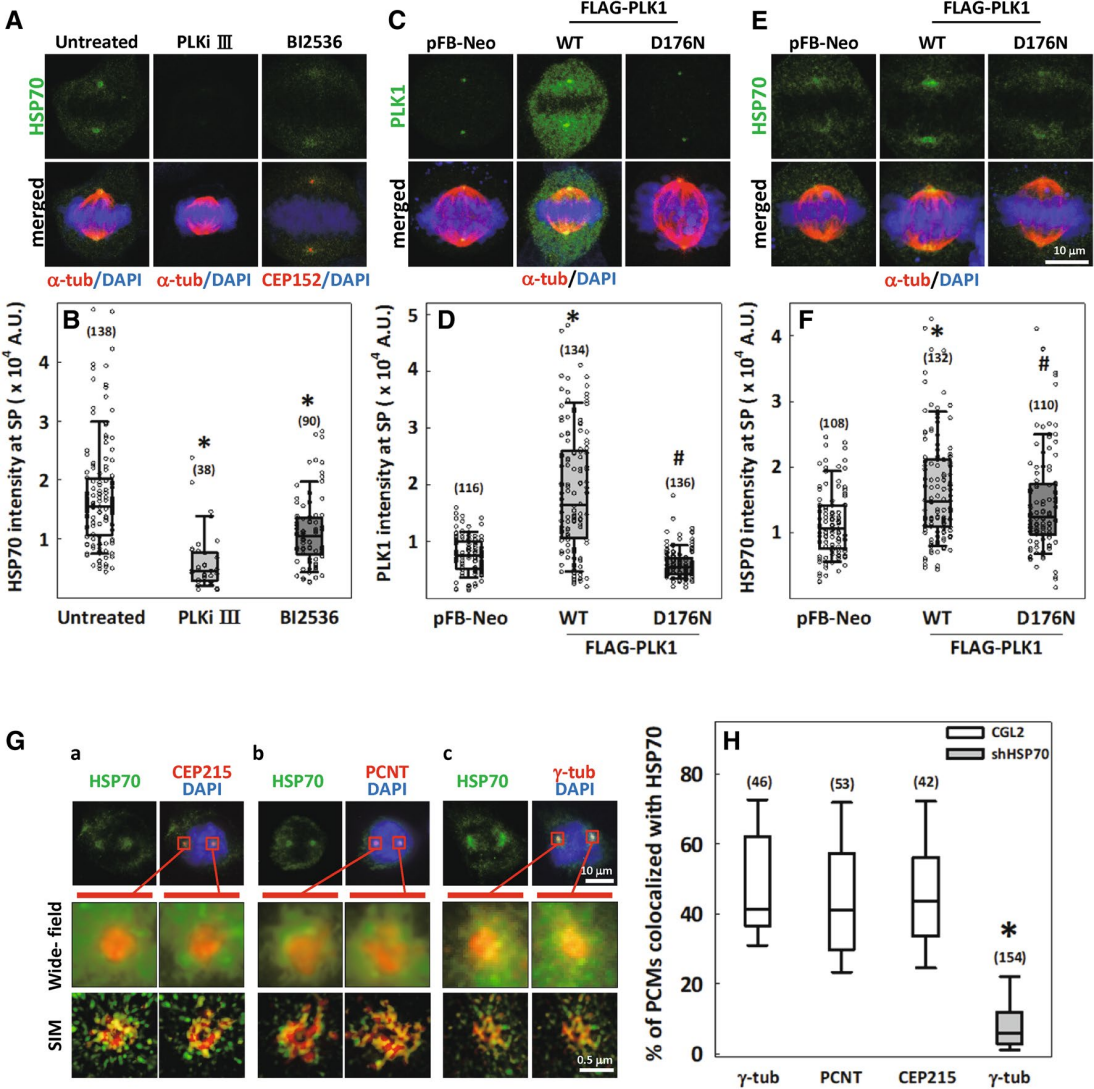
HSP70 accumulates at the spindle pole in a PLK1-dependent manner and colocalizes with PCNT, CEP215 and γ-tubulin. **A** Representative images of immunostained HSP70 (green) at the spindle pole in the CGL2 cells. Cells were treated with 20 μM PLK inhibitor III or BI2536 30 min before fixation. Spindle poles were identified by either α-tubulin (α-tub, red) or CEP152 (red) staining. **B** Relative HSP70 intensity at the spindle pole (SP) was measured. Box plots show the 10th, 25th, 50th, 75th, 90th percentiles, and numbers in parenthesis (n) indicate the number of spindle poles examined. *p < 0.05, significant decrease compared to untreated by Mann–Whitney rank sum test. **C**–**F** PLK1 (**C** green) and HSP70 (**E** green) accumulation at the spindle pole (specified by α-tubulin, red) in cells transduced with control vector (pFB-Neo), FLAG-PLK1-WT or D176N. **D**, **F** Relative intensity of PLK1 (**D**) and HSP70 (**F**) at the spindle pole (SP). Numbers of examined spindle poles (n) are indicated. Mann–Whitney rank sum test was performed. *p < 0.05 comparing WT to pFB-Neo; ^#^*p *< 0.05 comparing D176N to WT. **G** Colocalization of HSP70 (green) with CEP215 (a), PCNT (b) and γ-tubulin (c) (red) was revealed by 3D-SIM imaging. The mitotic centrosomes in the red-boxed regions in the upper panels were magnified and are presented as wide-field (middle) and SIM (bottom) images. **H** Percentages of each indicated PCM component that were colocalized with HSP70 with or without HSP70 depletion are shown in the box plot with the number of spindle poles (n) indicated. *p < 0.05 by Mann–Whitney rank sum test comparing shHSP70 to CGL2

Based on our previous study, which used immunofluorescence staining to reveal that HSP70 localizes to the spindle poles during mitosis [31], we first investigated how the localization of HSP70 at the spindle pole is regulated. Phosphorylation of HSP70 at various residues has been shown to regulate its function [27, 32, 33], so we screened a series of kinase inhibitors to test whether they may affect the accumulation of HSP70 at the spindle pole. The PLK inhibitor III (an inhibitor of PLK members) and BI2536 (an inhibitor of PLK1 [34]) dramatically reduced the intensity of HSP70 at the spindle poles (Fig. 1A, B). This result is consistent with previous studies utilizing MDA-231 and HeLa-S3 cells [28, 35]. Based on these findings and the previous report that HSP70 is a PLK1 substrate [33], we further examined the effects of ectopic overexpression of FLAG-tagged PLK1 on HSP70 accumulation at the spindle poles. Overexpression of WT-PLK1 dramatically increased the intensities of both PLK1 (Fig. 1C, D) and HSP70 (Fig. 1E, F) at the spindle poles. We also overexpressed a kinase-dead PLK1 with an Asp^176^ to Asn mutation (D176N) in the catalytic base that has been demonstrated to slow down PLK1 exchange at the centrosome and hence reduce its accumulation [20]. The kinase-dead PLK1 did not elevate the intensities of either PLK1 (Fig. 1C, D) or HSP70 (Fig. 1E, F) at the spindle poles to the level observed in WT-PLK1-overexpressing cells. These data indicate that HSP70 localizes to the spindle pole at least as a partial result of PLK1 kinase activities. We also examined the localization of HSP70 with 3D-SIM and dissected the colocalization of HSP70 with the centrosome markers including PCNT, CEP215 and γ-tubulin. HSP70 appeared as a mesh-like structure scattered around and often colocalized with PCM components, including PCNT (Fig. 1G-a), CEP215 (Fig. 1G-b) and γ-tubulin (Fig. 1G-c). Quantitative measurements from 3D-SIM images revealed that up to 40% of each PCM component colocalized with HSP70; moreover, depletion of HSP70 by shRNAs targeting *HSPA1A* and *HSPA1B* dramatically reduced the percentage of γ-tubulin colocalized with HSP70 (Fig. 1H). Together, these data indicate that PLK1, a master mitotic kinase [15, 16, 36], may positively regulate HSP70 spindle pole accumulation, and HSP70 may associate with PCNT, CEP215 and γ-tubulin at the spindle poles as a result of PLK1 kinase activity.

### Loss of HSP70 induces spindle pole fragmentation and disrupts the accumulation of PCNT and CEP215 at the mitotic centrosome

**Fig. 2:**
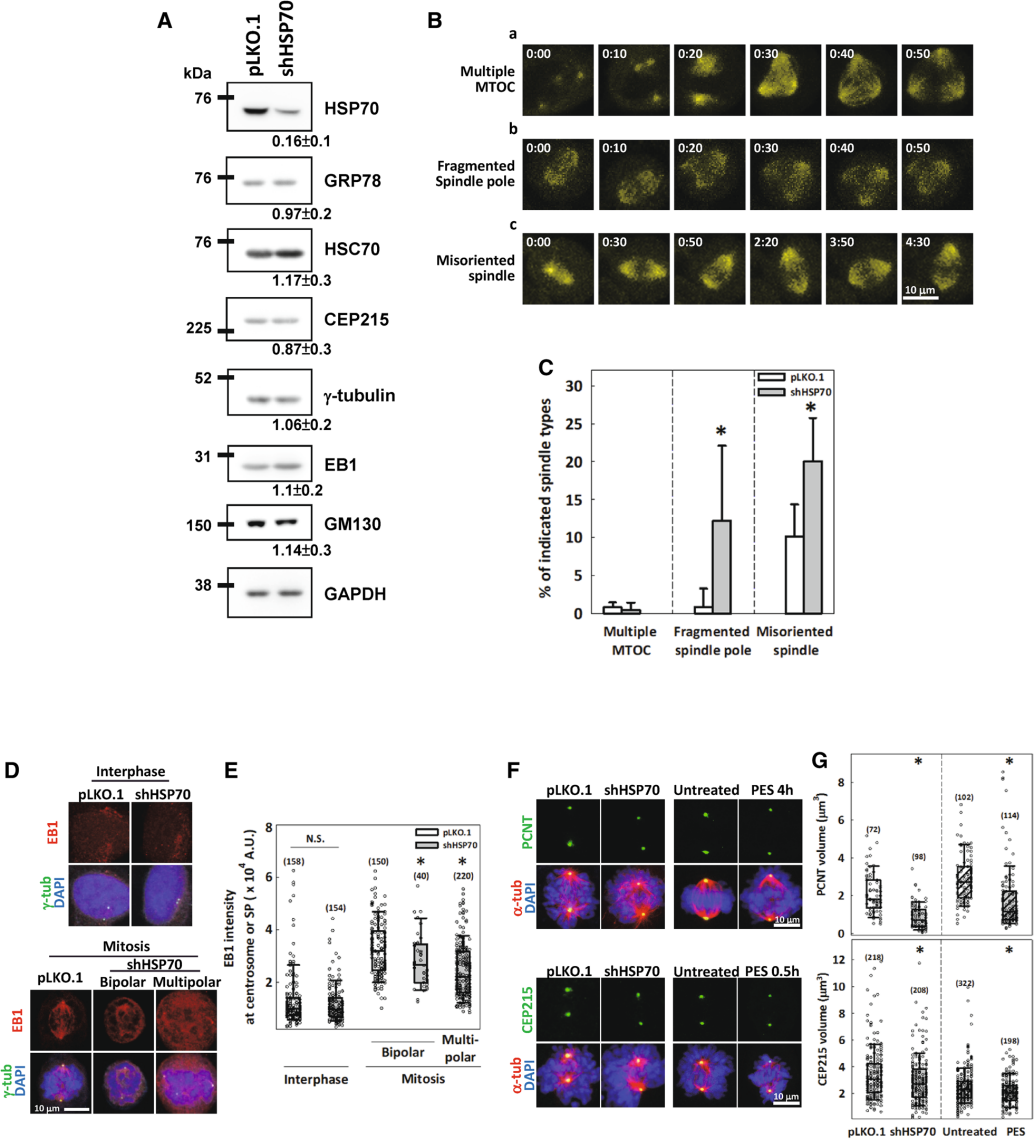
Loss of HSP70 function induces spindle pole fragmentation and disrupts the accumulation of PCNT and CEP215 at the mitotic centrosome. **A** Western blot analysis of the efficiency and specificity of shRNA-mediated depletion of HSP70. The numbers underneath each blot indicated the ratio of expression level of each protein to that in the pLKO.1 control as normalized by the loading control GAPDH. Mean ± SD from three independent experiments. **B** Types of abnormal spindle formation in HeLa-S3-tub-EYFP cells were visualized by time-lapse imaging, and still images from the indicated times are shown. (a) Cells with more than two visible MTOCs before mitosis entry were regarded as ‘multiple MTOC’. (b) Mitotic cells in which a bipolar spindle was first constructed, but then either pole was split into multiple poles in the later frames were defined as ‘fragmented spindle pole’. (c) Mitotic cells with bipolar spindles that did not split into extra poles but kept rotating the spindle axis with varying orientations throughout the imaging period were classified as ‘misoriented spindle’. **C** Percentages of control vector-transduced (pLKO.1) or HSP70-depleted (shHSP70) mitotic Hela-S3-tub-EYFP cells with the indicated types of abnormal spindles. *p < 0.05 by Mann–Whitney rank sum test comparing shHSP70 to pLKO.1. The percentage of mitotic cells containing abnormal mitotic spindles was determined using at least 100 mitotic cells from six independent experiments. **D** Representative images of control or HSP70-depleted interphase and mitotic CGL2 cells immunostained with EB1 (red) and γ-tubulin (γ-tub, green). **E** Relative EB1 intensity at the centrosome or spindle pole (SP) with number of spindle poles (n) indicated. Mann–Whitney rank sum test was performed. *p < 0.05 comparing shHSP70 to pLKO.1; N.S. no significance. **F** Representative images of control or HSP70-depleted and untreated or PES-treated CGL2 cells immunostained with PCNT or CEP215 (green). The spindle was revealed by α-tubulin immunostaining (α-tub, red). Cells were treated with 10 μM and 40 μM PES for 4 h and 0.5 h, respectively, before fixation. **G** Quantification of PCNT and CEP215 volumes at the spindle pole was performed, and values are shown in the box plots with the number of examined spindle poles (n) indicated. *p < 0.05 comparing shHSP70 to pLKO.1 or PES to untreated by Mann–Whitney rank sum test

HeLa-S3 cells stably expressing EYFP-fused tubulin were utilized to monitor the role of HSP70 in determining the function of mitotic centrosomes. The inducible form of HSP70 was depleted by the shRNAs targeting *HSPA1A* and *HSPA1B* genes as in the previous report [31] and the depletion specificity and efficiency were verified (Fig. 2A). HSP70 depletion did not increase the percentage of cells already containing multiple MTOCs upon entry into mitosis (Fig. 2B-a and C), suggesting that loss of HSP70 is not likely to induce centrosome over-duplication. However, HSP70 depletion dramatically increased the percentages of cells with spindle pole fragmentation and misoriented spindle after entering into mitosis (Fig. 2B-b, c and C), which are indicators of functional defects in the mitotic centrosome [37–39]. In addition, immunofluorescence staining revealed that HSP70 depletion dramatically decreased the intensity of EB1, a MT nucleation indicator [40], in the centrosomes of mitotic cells but not interphase cells (Fig. 2D, E). These results indicated that HSP70 depletion specifically disrupts the function of mitotic centrosomes and induces fragmentation of the spindle pole. Centrosome maturation is a prerequisite for enhanced MT nucleation from the mitotic centrosome, and PCNT and CEP215 are two proteins that are essential for maturation due to their accumulation at the maturing centrosome and recruitment of other necessary PCM components [3, 4]. Accordingly, we also observed dramatic reductions in the volumes of both proteins at the mitotic centrosome in cells depleted of HSP70 or treated with PES (Fig. 2F, G), an HSP70 inhibitor [31], suggesting that HSP70 is required for their accumulation. Since Western blot analysis indicated that the overall expression of EB1, γ-tubulin, and CEP215 was not affected by HSP70 depletion (Fig. 2A), these data suggest that HSP70 is required for the integrity and correct functioning of mitotic centrosomes, thus enabling MT nucleation and bipolar spindle assembly.

### HSP70 is required for the 3D assembly of PCNT, CEP215 and γ-tubulin at the mitotic centrosome

**Fig. 3:**
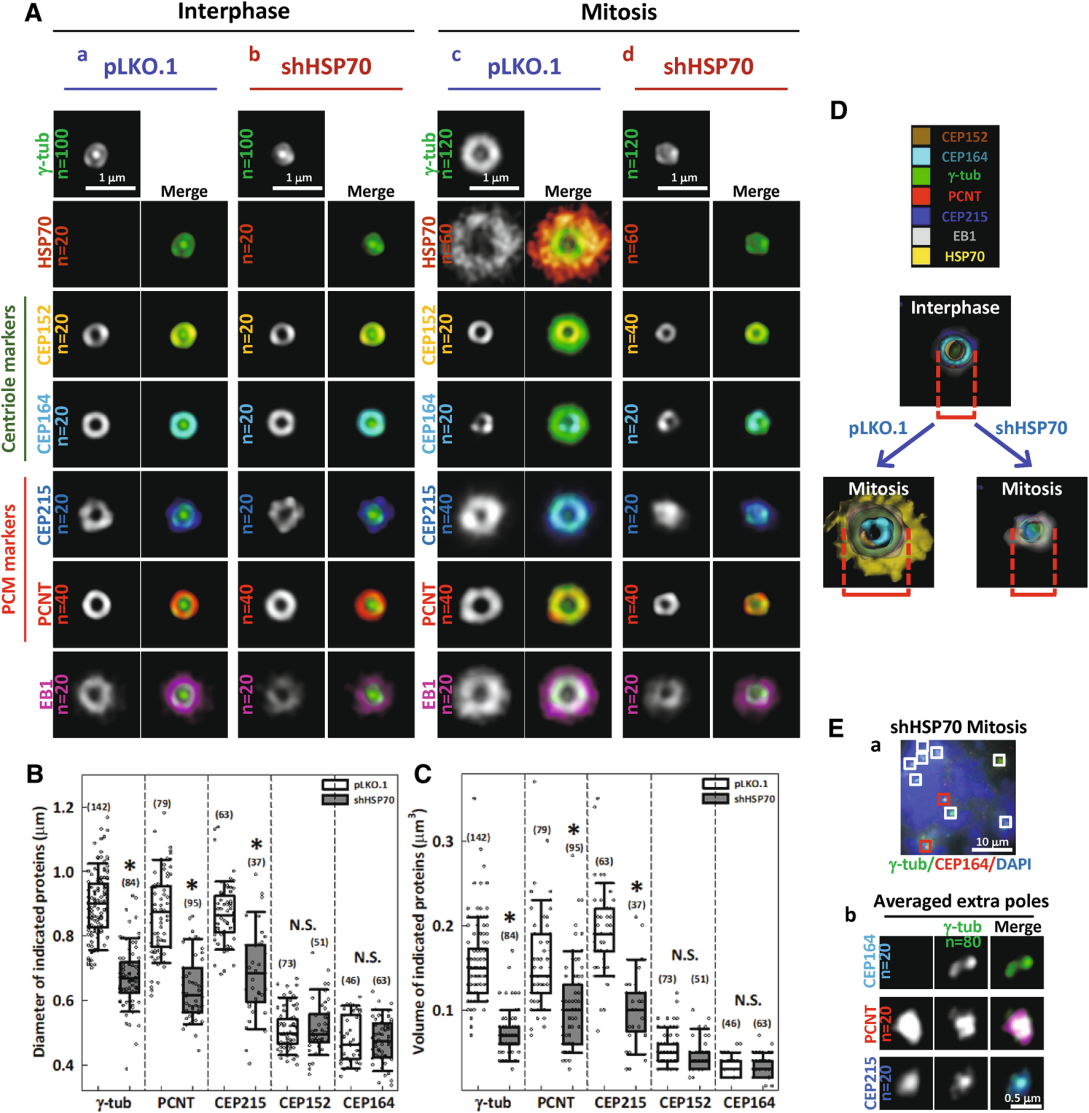
HSP70 is required for the 3D assembly of CEP215, PCNT and γ-tubulin at the mitotic centrosome. **A** 2D projections of the averaged 3D-SIM images of each indicated centrosome marker in control interphase (a) and mitotic cells (c) and HSP70-depleted interphase (b) and mitotic (d) cells. Merged images show the relative distribution of each centrosome marker with respect to γ-tubulin. **B**, **C** Box plots show the relative diameter and volume of each marker in control and HSP70-depleted mitotic cells with the number of samples (n) indicated. Mann–Whitney rank sum test was performed. *p < 0.05 comparing shHSP70 to pLKO.1. **D** Surface-rendered images of the centrosome with or without HSP70. **E** (a) Representative wide-field image of a HSP70-depleted mitotic cell with multipolar spindle. Two poles with centriole marker (CEP164) are indicated with red boxes, and the other extra spindle poles without CEP164 are indicated by white boxes. (b) Averaged SIM images of the extra spindle poles

To further characterize the effects of HSP70 on PCNT, CEP215 and γ-tubulin accumulation at the mitotic centrosome, we analyzed the distribution of each protein at the spindle poles by 3D-SIM imaging in control and HSP70-depleted cells. We co-stained samples for PCM components and γ-tubulin, which served as a reference for 3D volume alignment and averaging by Chimera software [11, 41]. This strategy for processing images allowed us to compare the overall structure of each averaged centrosome marker with or without HSP70. CEP164, a distal appendage marker at the mother centriole, and CEP152, an inner PCM component that proximally surrounds the centrioles [9, 10, 42], were also stained to reveal the orientation of the centrosomes. The spatial distribution of HSP70 staining with respect to the centrosome markers was also assessed. Figure 3A shows the 2D projections of averaged 3D-SIM centrosome images. Figure 3A-a shows that in the centrosomes of control interphase cells, CEP164 and CEP152, γ-tubulin, PCNT and CEP215, were all arranged in a concentric toroid distribution, while γ-tubulin appeared as a dot at the center of the ring, as reported previously [9, 10]. EB1 appeared as a slightly larger ring immediately surrounding γ-tubulin. Little or no HSP70 appeared at the interphase centrosome. At the mitotic centrosome of control cells, shown in Fig. 3A-c, the distribution of CEP152 and CEP164 remained similar to that of the interphase centrosome, while γ-tubulin, PCNT and CEP215 were accumulated and expanded into a larger ring structure than was observed in the interphase centrosomes, consistent with current knowledge [3]. Importantly, HSP70 signal was dramatically increased at and surrounded all the other stained PCM components, suggesting a robust recruitment of HSP70 to the mitotic centrosome. Additionally, the EB1 ring was dramatically enlarged, suggesting enhanced MT nucleation capacity. In the HSP70-depleted interphase cells (Fig. 3A-b), all the centrosome markers appeared structurally similar to those in control interphase cells, suggesting that loss of HSP70 did not significantly alter their distribution during interphase. However, in the HSP70-depleted mitotic cells (Fig. 3A-d), γ-tubulin, PCNT and CEP215 were not expanded to the level observed in control mitotic cells. Interestingly, CEP152 and CEP164 signals were not significantly different between HSP70-depleted and control mitotic cells. Quantitative analysis revealed that the diameters and volumes of PCNT, CEP215 and γ-tubulin signals, but not CEP152 and CEP164 signals, were significantly reduced in the HSP70-depleted mitotic cells (Fig. 3B, C). Figure 3D shows the surface rendered image of all the stained markers and reveals a concentric toroid structure of the mitotic centrosomes in control cells (lower left) and a severely altered structure of mitotic centrosomes in HSP70-depleted cells (lower right). Since depletion of HSP70 did not significantly alter the expression levels of CEP215, γ-tubulin, and EB1 (Fig. 2A), these data suggest that HSP70 is required for proper 3D assembly of PCNT, CEP215 and γ-tubulin. Notably, in the multipolar spindle induced by HSP70 depletion, the centriole markers (CEP152 or CEP164) were found in only two of the poles (Fig. 3E-a, red box), but the PCM components (PCNT or CEP215) were observed in all the multiple poles (Fig. 3E-a, white box and Fig. 3E-b). This observation suggests that the extra poles might arise from fragmentation of the compromised mitotic centrosome, implicating HSP70 as an important factor for maintaining the integrity of the spindle pole. Together, the subdiffraction resolution images suggest that HSP70 is a crucial promoter of PCM assembly at the mitotic centrosome, and loss of HSP70 significantly disrupts their assembly, and thus MT nucleation.

### HSP70 is required for the interaction between PCNT and CEP215 at the mitotic centrosome

**Fig. 4:**
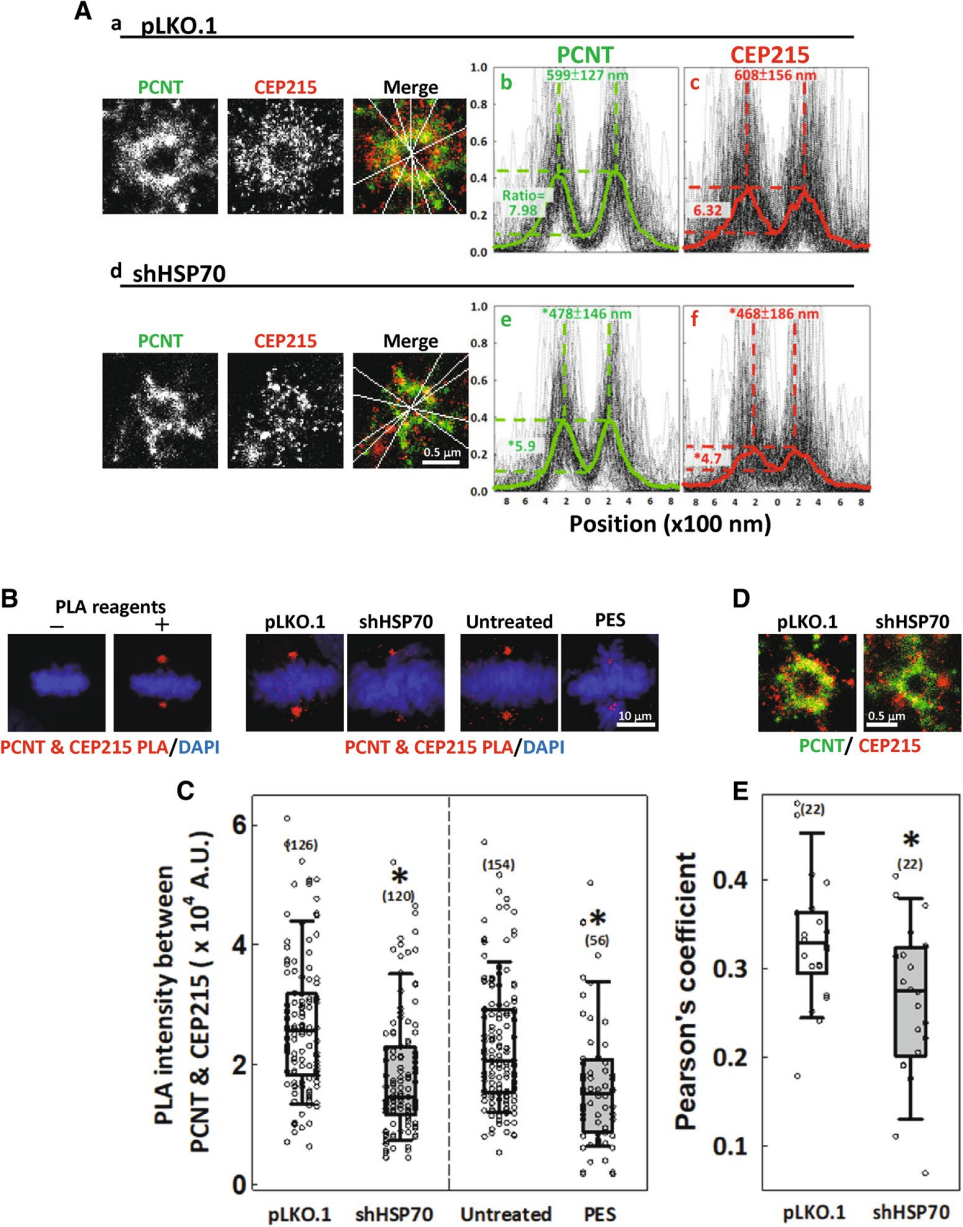
HSP70 is required for the interaction between PCNT and CEP215. **A** GSD images of PCNT (green) and CEP215 (red) in control (a) and HSP70-depleted (d) mitotic cells. Lines of different directions across the center are shown in merged images. (b, c, e and f) The intensities along each line within a central 1.8-μm region were plotted. At least 20 spindle poles were analyzed and five lines were drawn in each pole. Mean distribution curves are shown in green for PCNT and red for CEP215. The distance between two peak regions (mean ± SD) and the peak-center intensity ratio (median), which were determined as described, are presented and were tested by Student’s *t* test and Mann–Whitney rank sum test, respectively. *p < 0.05 comparing shHSP70 to pLKO.1. **B** PLA was performed as described in “Methods” to measure the interaction between PCNT and CEP215. Left, representative images of PLA signal generated using heat-inactivated (−) or normal activity (+) PLA reagents. Right, representative confocal images of cells depleted of HSP70 or treated with 40 μM PES 30 min before fixation are shown. **C** Relative PCNT and CEP215 PLA intensity at the spindle pole with the number of examined spindle poles (n) indicated. Mann–Whitney rank sum test was performed. *p < 0.05 comparing shHSP70 to pLKO.1 or PES to untreated. **D** Representative GSD images of colocalization between PCNT (green) and CEP215 (red) at the spindle pole of mitotic cells from control or HSP70-depleted cultures. **E** The level of PCNT and CEP215 colocalization was measured by Pearson’s correlation coefficient, and the coefficient ranking is shown with the number of examined spindle poles (n) indicated. Mann–Whitney rank sum test was performed. *p < 0.05 comparing shHSP70 to pLKO.1

To confirm the roles of HSP70 in the mitotic centrosome assembly, we also performed two-color GSD superresolution imaging to examine the distribution of PCNT and CEP215 at the mitotic centrosome. GSD images showed that in the control mitotic cells, both PCNT and CEP215 molecules accumulate and distribute in a well-expanded toroid structure (Fig. 4A-a). However, in the HSP70-depleted cells, neither protein formed the expanded toroid structure observed in control cells (Fig. 4A-d). The distribution of PCNT and CEP215 was assessed by line profile analysis of the fluorescence intensities across the center of the toroid. The average distribution of each protein revealed two regions of highest fluorescence intensity that flanked the central region (Fig. 4A-b and -c), reflecting the toroidal distribution of both proteins. The distance between two peak regions may indicate the diameter of the toroid, and the ratio of intensities at the peaks to that at center region may indicate the extent to which the proteins accumulated. The distance between the two peak regions were 599 ± 127 nm and 608 ± 156 nm, and the peak-center intensity ratios were 7.98 and 6.32 for PCNT and CEP215, respectively, in the mitotic centrosome of control cells. In HSP70-depleted cells, however, the distance between the two peaks were significantly decreased to 478 ± 146 and 468 ± 186, and the peak-center intensity ratios were also reduced at 5.9 and 4.7 for PCNT and CEP215, respectively (Fig. 4A-e and -f), implying considerable disruption of PCNT and CEP215 accumulation and expansion in HSP70-depleted cells. The results from GSD imaging also supported the notion that HSP70 facilitates the assembly of PCM components at the mitotic centrosome. During centrosome maturation, PCNT and CEP215 are known to physically interact with each other, forming a complex that provides a proper environment for recruiting other PCM components [17]. To further confirm the requirements of HSP70 in centrosome maturation, we utilized a proximity ligation assay (PLA)—a commonly used technique for detecting protein–protein interactions within a range of 40 nm [43]—to test whether the interaction between PCNT and CEP215 occurs with or without HSP70. A PLA signal between PCNT and CPE215 was detected specifically at the centrosome, with each interphase cell exhibiting 1 or 2 foci (data not shown) and mitotic cells routinely containing 2 foci at the spindle poles (Fig. 4B, left panel +), whereas PLA procedure using heat-inactivated reagents yielded no clear signals (Fig. 4B, left panel −). Confocal images and the quantified signal from the PLA revealed that HSP70 depletion or application of PES significantly reduced the intensity of the PLA signal (Fig. 4B right panel and C), suggesting that HSP70 is required for the proper interaction between PCNT and CEP215. The interaction between PCNT and CEP215 was also assessed by colocalization analysis from GSD images (Fig. 4D). Colocalization of PCNT and CEP215 was evaluated according to Pearson’s correlation coefficient, which was significantly lower in HSP70-depleted cells than controls (Fig. 4E), further supporting the importance of HSP70 in the interaction between PCNT and CEP215.

### HSP70 facilitates PLK1 accumulation at the spindle pole and ameliorates PLK1 interference-induced spindle abnormalities

**Fig. 5:**
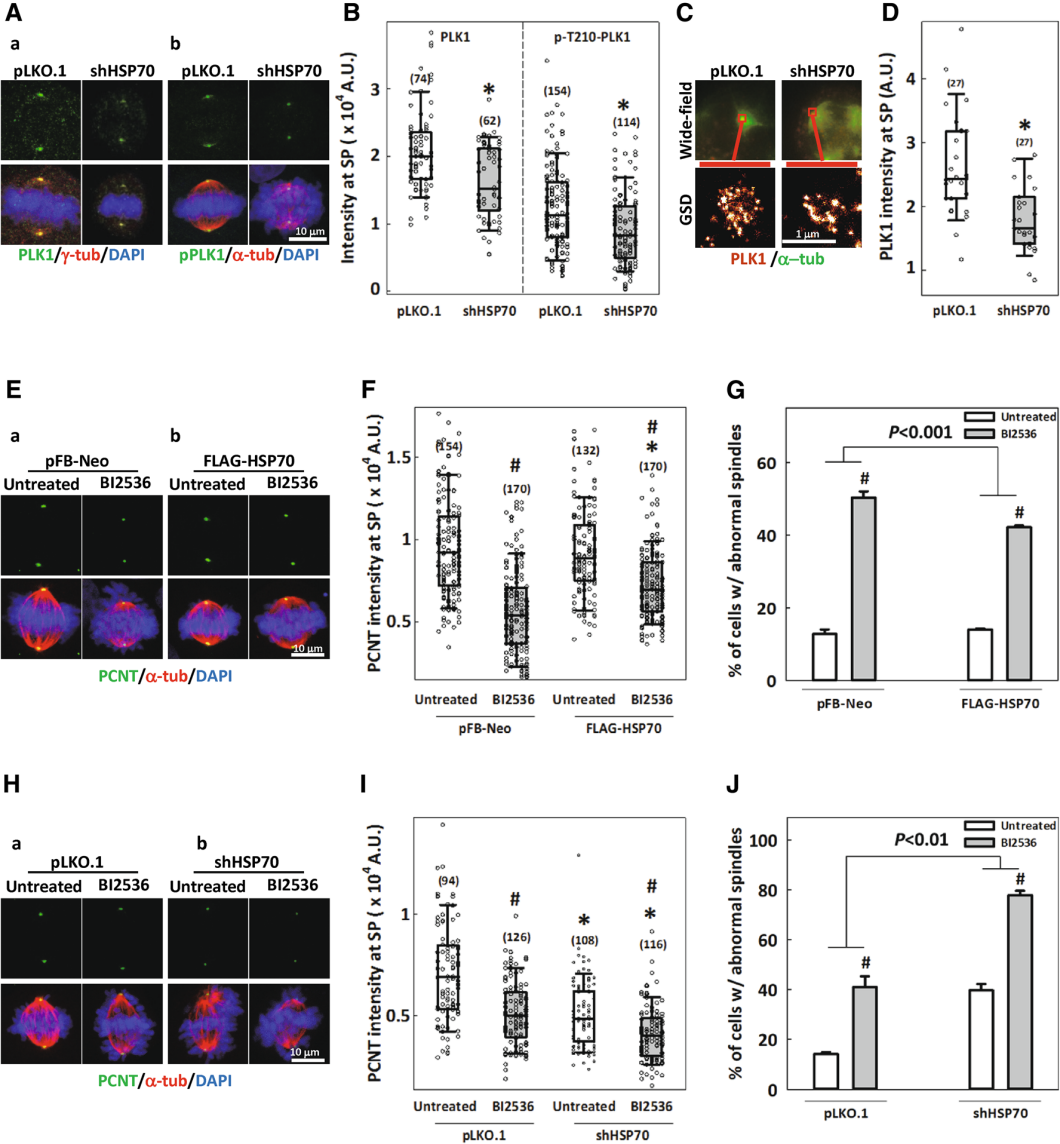
HSP70 facilitates PLK1 accumulation at the spindle pole and ameliorates PLK1 interference-induced spindle abnormalities. **A** Representative images of PLK1 or p-T210-PLK1 (green) at the spindle pole specified by γ- or α-tubulin (red) in pLKO.1- or shHSP70-transduced cells. **B** Relative PLK1 and p-T210-PLK1 intensity at the spindle pole (SP) with numbers of examined spindle poles (n) indicated. Mann–Whitney rank sum test was performed. *p < 0.05 comparing shHSP70 to pLKO.1. **C** GSD images of PLK1 accumulation at the spindle pole, specified by α-tubulin (α-tub, green), in control or HSP70-depleted cells. Spindle pole regions in the wide-field images are indicated with red boxes (upper panel), and magnifications are shown in the GSD images (lower panel). **D** PLK1 intensity at the spindle pole (SP) was determined from GSD images and numbers of examined spindle poles (n) are indicated. Mann–Whitney rank sum test was performed. *p < 0.05 comparing shHSP70 to pLKO.1. **E** Representative images of pFB-Neo—(a) and FLAG-HSP70—(b) transduced cells, untreated or treated with 10 μM BI2536 for 30 min before fixation, and immunostained with PCNT (green) and α-tubulin (red). **F** Relative PCNT intensity at the spindle pole (SP) with numbers of examined spindle poles (n) indicated. Mann–Whitney rank sum test was performed. ^#^*p *< 0.05 comparing BI2536 to untreated; *p < 0.05 comparing FLAG-HSP70 to pFB-Neo. **G** Percentages of cells with abnormal spindles. ^#^*p *< 0.05 comparing BI2536 to untreated by Student’s *t* test; *p* value is indicated comparing FLAG-HSP70 to pFB-Neo by two-way ANOVA. **H** Representative images of pLKO.1—(a) and shHSP70—(b) transduced cells, untreated or treated with 10 μM BI2536 for 30 min before fixation, and immunostained with PCNT (green) and α-tubulin (red). **I** Relative PCNT intensity at the spindle pole (SP) with the number of examined spindle poles (n) indicated. Mann–Whitney rank sum test was performed. ^#^*p *< 0.05 comparing BI2536 to untreated; *p < 0.05 comparing shHSP70 to pLKO.1. **J** Percentages of cells with abnormal spindles. ^#^*p *< 0.05 comparing BI2536 to untreated by Student’s *t* test; *p* value is indicated comparing shHSP70 to pLKO.1 by two-way ANOVA

PLK1 has been proposed to phosphorylate numerous PCM components and drive their accumulation at the centrosome to promote centrosome maturation [4, 14–16, 44]. Figure 5A and B show that both PLK1 and its mitotically activated form, phospho-T210-PLK1 [45, 46], localized to the spindle poles in control cells, but the accumulation at this location was significantly reduced in HSP70-depleted cells. We also examined PLK1 recruitment by GSD imaging, which allowed us to measure the relative quantities of the immunostained proteins. The results showed that PLK1 accumulation at the spindle pole was significantly lower in the HSP70-depleted cells compared to controls (Fig. 5C, D), confirming that the loss of HSP70 disrupts PLK1 recruitment. These findings suggest that in addition to being downstream of PLK1, HSP70 is also required for PLK1 accumulation at the spindle poles. In order to confirm the role of HSP70 as a mediator of PLK1 function during mitosis, we modulated the cellular level of HSP70 and examined the effects on PLK1 inhibition-induced abnormalities in the spindle and mitotic centrosome. In cells transduced with empty vector pFB-Neo, treatment of the PLK1 inhibitor, BI2536, significantly reduced PCNT intensity at the spindle poles and induced the formation of abnormal spindles (Fig. 5E-a, F, G). Overexpression of FLAG-HSP70 did not change PCNT intensity or the percentage of abnormal spindles in untreated cells, but it partially restored the BI2536-induced reduction of PCNT intensity and ameliorated BI2536-induced abnormal spindle formation (Fig. 5E-b, F, G). Since HSP70 overexpression could partially rescue mitotic defects induced by PLK1 inhibition, we then proceeded to examine the effects of HSP70 depletion on the BI2536-induced spindle abnormalities. Not surprisingly, BI2536 treatment dramatically reduced the PCNT intensity and induced abnormal spindle formation in cells transduced with empty vector pLKO.1 (Fig. 5H-a, I, J), while HSP70 depletion reduced PCNT intensity and induced abnormal spindle formation as compared to the control depleted cells (Figs. 2E, F and 5H–J). Importantly, BI2536-induced reduction of PCNT intensity and formation of abnormal spindles were enhanced in the HSP70-depleted cells, suggesting that loss of HSP70 exacerbated the mitotic abnormalities induced by PLK1 inhibition. Together, these data further support the notion that HSP70 mediates PLK1 effects during mitosis to support centrosome and spindle assembly.

### HSPA1A chaperone activity is required for PCNT accumulation at the mitotic centrosome and assembly of mitotic spindles

**Fig. 6:**
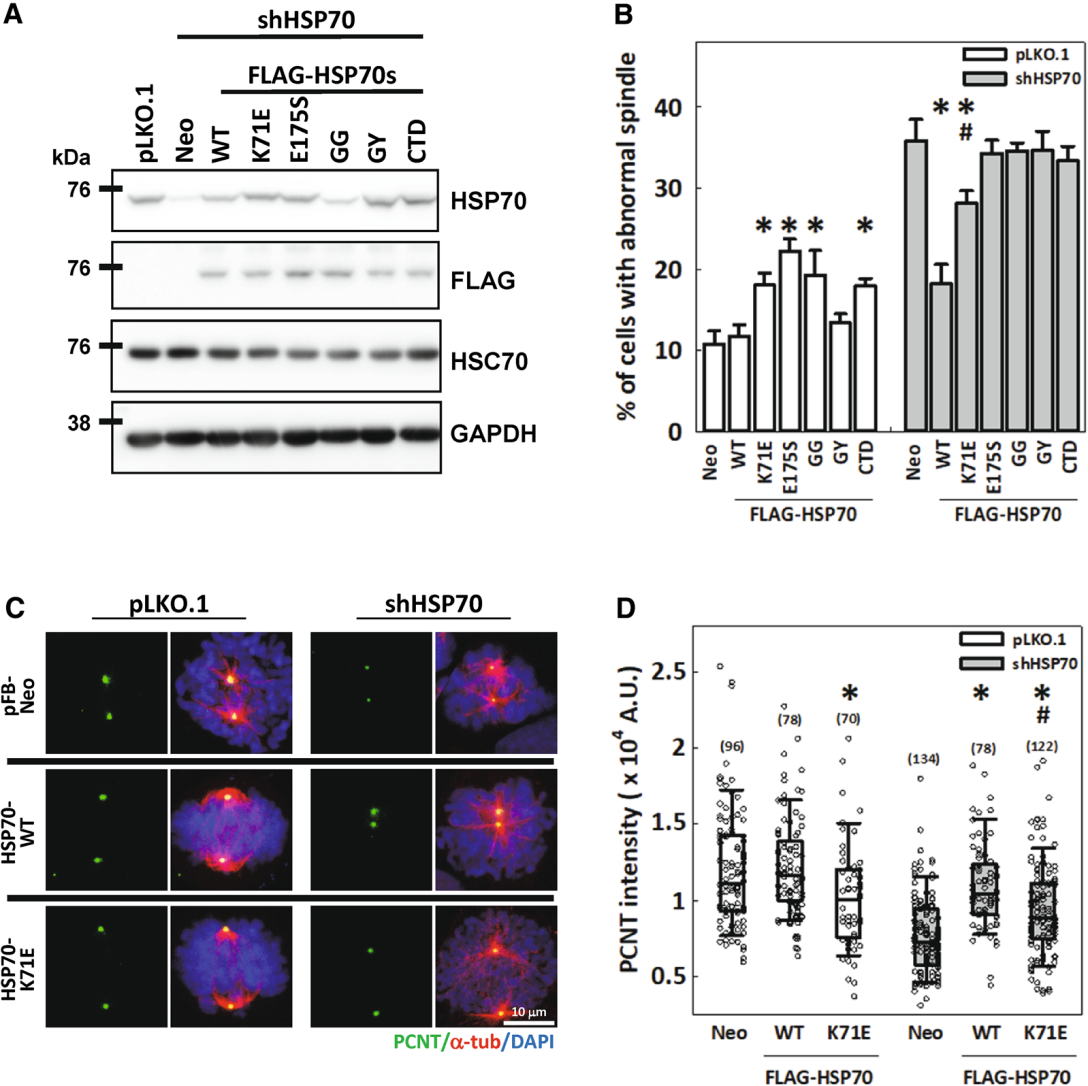
HSPA1A chaperone activity is required for PCNT accumulation at the mitotic centrosome and assembly of mitotic spindles. **A** Western blot analysis of the expression level of FLAG-tagged wild type and each mutant HSP70. **B** CGL2 cells stably expressing FLAG-HSP70s were depleted of endogenous HSP70, and then the cells were immunostained with PCNT and α-tubulin to reveal the spindle. The percentages of cells with abnormal spindles were determined by at least 600 mitotic cells from three independent experiments, and Student’s *t* test was performed. *p < 0.05 comparing FLAG-HSPA1As to pFB-Neo (Neo); ^#^*p *< 0.05 comparing mutants to WT. **C** Representative images of PCNT (green) and spindles (α-tubulin, red). **D** Relative PCNT intensity at the spindle pole (SP). Numbers in parenthesis indicate the number of spindle poles examined. Mann–Whitney rank sum test was performed. *p < 0.05 comparing FLAG-HSPA1As to pFB-Neo (Neo); ^#^*p *< 0.05 comparing K71E to WT

HSP70 family members are multidomain proteins consisting of a nucleotide binding domain (NBD) that regulates the binding of either ATP or ADP, a substrate-binding domain (SBD) for processing client proteins, and a C-terminal-IEEVD motif for interaction with the tetratricopeptide repeat-containing co-chaperones [47, 48]. In addition to preventing client proteins from misfolding by direct binding (holding) of the SBD, the ATP-ADP cycling of the NBD also allows HSP70 to further unfold, refold or disaggregate client proteins, providing the chaperones with multi-layered activities [25, 49]. To dissect the specific HSP70 function that regulates the mitotic centrosome, two mutations at the ATPase domain of HSP70 (K71E and E175S), two double mutations in the SBD [A406G + V438G (GG) and A406G + V438Y (GY)], as well as a C-terminal-IEEVD deletion mutant (CTD), were created from a FLAG-tagged HSPA1A construct [50–52]. These mutants were then expressed in cells, and the effects on mitotic spindle assembly were assessed. The expression levels of these recombinant proteins were relatively corresponding and had little effect on HSC70 expression as examined by Western blotting (Fig. 6A). Expression of either HSP70-K71E (an ATPase-dead mutant that retains substrate-holding activity) or E175S (a mutant locked in an ADP-bound conformation and therefore lacking both ATPase and refolding activities) induced the assembly of the abnormal mitotic spindles. Expression of HSP70-GG (a mutant that can still bind substrates but cannot process them completely) also induced abnormal spindles, whereas expression of HSP70-GY (a null mutant with thoroughly disrupted hydrophobic pockets in the SBD that cannot bind substrates) did not significantly induce abnormal spindle formation. Furthermore, expression of HSP70-CTD also induced abnormal spindle formation (Fig. 6B). These data suggest that HSP70-K71E, -E175S, -GG, and -CTD may have dominant-negative effects on client proteins and/or cofactors that are required for precise assembly of mitotic spindles. We then proceeded to examine the effects of the mutants in cells depleted of endogenous HSP70. Expression of HSP70-WT significantly ameliorated the formation of abnormal mitotic spindles in cells depleted of endogenous HSP70, and HSP70-K71E also suppressed abnormal spindle formation, although to a lesser extent. On the contrary, HSP70-E175S, -GG, -GY, and -CTD showed no rescue effects (Fig. 6B). These observations suggest that the substrate binding/holding activity (independent of ATPase activity) of HSP70 could promote bipolar spindle assembly, while the ATPase-dependent substrate binding and processing activity of HSP70 and its interaction with co-chaperones are required for fully functional regulation of spindle assembly. In addition, PCNT accumulation at the mitotic centrosome was decreased in cells overexpressing HSP70-K71E compared with those transduced with empty vector (Fig. 6C, D), indicating that the overexpressed HSP70-K71E might compete with the endogenous HSP70 hence impair PCNT recruitment to the mitotic centrosome. Furthermore, the HSP70 depletion-induced reduction in PCNT intensity could be fully restored by HSP70-WT but was only partially restored by HSP70-K71E (Fig. 6B, C), suggesting that HSP70-mediated PCNT accumulation at the mitotic centrosome also depends on its ATPase-independent and -dependent chaperone activities and may be responsible for the rescue effects of HSP70 on the assembly of mitotic spindle. Together, these data indicate that the ATPase-independent substrate binding, ATPase-dependent folding/refolding, and/or co-chaperone binding activities of HSP70 are all required for the proper assembly of mitotic centrosomes and the subsequent mitotic spindle.

## Discussion

The accumulation of the PCM components at the centrosome during maturation is a delicately regulated process that is essential for the ability of the mitotic centrosome to enhance MT nucleation and assemble a well-functioning bipolar spindle. In this study, we reveal that HSP70 associates with critical PCM components, including PCNT, CEP215 and γ-tubulin, to ensure appropriate 3D assembly of the mitotic centrosome and permit subsequent MT nucleation and bipolar spindle assembly.

The importance of HSP70 in the function and integrity of mitotic centrosomes and in mitosis progression has long been recognized. For example, Sse1, a member of the yeast HSP70 family, was reported to modulate the oligomeric state of the MT motor Cin8 to regulate the length of mitotic spindle [26]. Furthermore, NEK6 phosphorylates HSP70 and targets it to the mitotic spindle to facilitate chTOG–TACC3 complex recruitment, thus promoting kinetochore microtubule stability [27]. Overexpressed HSP70 was also shown to repair the heat shock-induced defects in the mitotic centrosome and facilitate subsequent progression through mitosis [29]. Additionally, HSP70 was shown to accumulate at the centrosomes of the heat shocked cells and prevent the proteasomal degradation of centrosome proteins [30]. These studies all suggested the possibility that HSP70 may regulate centrosome functions to support the mitotic progression, and we previously confirmed that HSP70 localizes to the mitotic spindle pole and is required for centrosome integrity, MT nucleation and spindle assembly [31]. However, the question of how HSP70 regulates the functions of the mitotic centrosome remained unanswered. In this study, we utilized the enhanced resolution of 3D-SIM and GSD microscopy to demonstrate that HSP70 colocalizes with PCM components, including PCNT, CEP215 and γ-tubulin. Loss of HSP70 significantly reduces the recruitment of these PCM components and disrupts their 3D assembly at the mitotic centrosome, leading to decreased MT nucleation. Since the proper functioning of the mitotic centrosome requires PCM components to be recruited in a highly organized manner, our results reveal that HSP70 permits MT nucleation and bipolar spindle assembly by promoting the recruitment and accurate 3D assembly of PCM components at the mitotic centrosome.

It has been shown that the *Hspa1a*^−*/*−^*/Hspa1b*^−*/*−^ double knock out (KO) mice can grow to adulthood with only mild cardiac hypertrophy [53], indicating that HSP70 may be not essential for cell cycle progression and division during normal mouse embryo development. However, the early-stage embryonic cells exhibit a very rapid cell cycle progression and their divisions involve a gradual transition from the acentrosomal spindle assembly to the centrosomal spindle assembly during embryo development [54–56]. These findings indicate that the embryonic cells might control the cell cycle progression, centrosome organization, and spindle assembly during development in ways different from those in differentiated somatic cells and thus might not depend on HSP70. Alternatively, it has been shown that the embryonic mouse hearts exhibit a robust regeneration after ablation of up to 60% of cardiac progenitor cells at embryonic day 7.5, which permits embryo development and survival [57]. Thus, the injuries and cell loss caused by HSP70 KO might induce a compensatory proliferation of fetal cardiomyocytes and the animal could eventually tolerate and adapt to HSP70 deficiency. Another study showed that the *Hspa1a*^−*/*−^*/Hspa1b*^−*/*−^ mice were on average 12% lighter in weight than wild-type newborns and had elevated levels of spontaneous genomic instability [58]. The lighter weight of the *Hspa1a*^−*/*−^*/Hspa1b*^−*/*−^ mice could result from reduced cell growth or cell loss as the authors demonstrated that the embryonic fibroblasts (MEF) prepared from these mice exhibited decreased cell growth and enhanced genomic instability. Our previous study [31] has demonstrated that inhibition of HSP70 induced mitotic arrest and reduced cell viability. In the current study, the malfunctioning mitotic centrosomes or the multipolar spindles induced in HSP70-depleted cells might initiate cell cycle arrest and/or cell death, which may also result in reduced cell growth or cell loss. Additionally, the MEFs from the mutant mice exhibited enhanced genomic instability, elevated micronuclei formation and chromosome damages, all of which could result from mitotic errors [59, 60]. Thus, the phenotypes exhibited in *Hspa1a*^−*/*−^*/a1b*^−*/*−^ mice could be partially explained by the effects of HSP70 depletion-induced defects on mitotic centrosomes and mitosis.

Recently, the NEK6-HSP70 axis was reported to facilitate centrosome clustering in cancer cells with amplified centrosomes, promoting mitotic progression through the formation of a pseudobipolar spindle [28]. Moreover, cancer cells with supernumerary centrosomes, which enter mitosis with multiple MTOCs, fail to resolve the multipolar spindle into a pseudobipolar spindle under HSP70 inhibition. Together with the previously reported function of NEK6-HSP70 in the recruitment of the chTOG–TACC3 complex, these data point to a role for HSP70 in regulating MT dynamics to facilitate centrosome clustering and bipolar division. In our study using an immortalized cell line, loss of HSP70 impaired the 3D structure of mitotic centrosomes and reduced PCM accumulation, an effect that was accompanied by the formation of extra spindle poles comprising disorganized PCM components. Thus, HSP70 depletion may specifically disrupt the stability of protein-rich PCM networks, and the PCM components that fail to be properly organized at the mitotic centrosome may contribute to new MT nucleating centers, leading to multipolar spindle formation. Interestingly, in the live imaging of mitotic progression in HSP70-depleted cells, we observed spindle pole fragmentation, which supports this hypothesis. Thus, in addition to its roles in regulating MT dynamics and centrosome clustering, our data provide another possible function of HSP70 in maintaining centrosome integrity and spindle bipolarity. As such, HSP70 appears to directly regulate the organization of mitotic PCM components, thus supporting their assembly in a bipolar spindle.

PCNT and CEP215 are putatively considered to be scaffold proteins based to their large sizes and predicted coil-coiled domains. Moreover, these proteins were proposed to physically interact with each other and polymerize around the maturing centrosome to form a matrix structure [17, 61, 62]. In drosophila, polo kinase, the PLK1 homolog, phosphorylates centrosomin, the CEP215 homolog, driving its structural rearrangement and assembly into a matrix structure; whereas in mammalian cells, the phosphorylation of PCNT and presumably CEP215 by PLK1 is prerequisite for the further recruitment of PCM components, promoting centrosome maturation [12, 15, 17, 19]. Based on these reports, it is currently believed that the recruitment of PLK1, PCNT and CEP215 to the centrosome is an interdependent process that occurs at the early phase of centrosome maturation [3–5]. Importantly, recruitment of PCM components is highly dynamic. PLK1 activity persists throughout mitosis and drives the continuous exchange of proteins, which are thought to be first incorporated in close vicinity to the centriole and then gradually extended outward into the PCM matrix [8, 12, 13, 20, 21, 63]. HSP70 has been proposed to be a mitotic substrate of PLK1 [33], which together with its observed localization at the spindle pole, implied a potential role for HSP70 in regulating centrosome maturation. Our results show that PLK1 may positively regulate HSP70 spindle pole accumulation, consisting with a previous report [28]. Intriguingly, we also observed that loss of HSP70 reduced PLK1 accumulation at the spindle pole and exacerbated mitotic defects induced by PLK1 inhibition, in addition to diminishing the PCNT-CEP215 interaction and accumulation at the spindle pole of the mitotic cells that had not yet formed multipolar spindles. These results suggest that HSP70 is recruited to the spindle pole by PLK1 and is also required for PLK1 recruitment and function at the spindle pole. We therefore hypothesize that HSP70 is a critical constituent of the maturing centrosome that cooperates with PLK1, PCNT and CEP215 to establish a functional mitotic centrosome in CGL2 cells.

The effects of the mutant HSP70s in spindle assembly suggest that HSP70 may regulate the accumulation of PLK1, PCNT and CEP215 through its chaperone activities, including prevention of substrate protein aggregation by direct binding (holding), ATP-fueled unfolding/refolding of polypeptides to regulate (alter)natively folded or oligomeric states, and the timely targeting of the substrate proteins for proteasomal or autophagic degradation [25, 32, 49, 64, 65]. In our study, HSP70-GG and -GY, two HSP70 mutants with disrupted SBD, failed to rescue the spindle defects induced by depletion of endogenous HSP70, implying the importance of the substrate binding in this process. Additionally, HSP70-K71E, which has disrupted ATPase activity but contains intact ability to bind substrates, showed a partial rescue effect, while HSP70-E175S, which is locked in an ADP-bound conformation and therefore lacks both ATPase and refolding activities, exhibited no rescue effect. These results imply that in addition to the substrate binding/holding activity of HSP70, ATP-ADP exchange is also required, and therefore, the repetitive refolding of substrates by HSP70 is likely to be required for a fully functioning mitotic centrosome. We hypothesize that during centrosome maturation, when abundant PCM components accumulate and form a highly protein-rich environment, HSP70 holding activities may provide a ‘buffering effect’ by maintaining certain PCM proteins in a properly folded state, preventing them from misfolding and aggregation. Concomitantly, HSP70 actions also require ATP-ADP cycling, which fuels repetitive processing of substrates to support the local transportation, oligomerization or interactions of the PCM proteins. This regulation of PCM components further supports the maturation and function of the mitotic centrosome. PCNT and CEP215, both of which play critical roles in centrosome maturation, tend to form polymerized complexes, and their homologs in drosophila were proposed to adopt specific conformations around the centrosome [11, 19]. Therefore, HSP70 could potentially maintain the proper conformation of these complexes via its client holding activity. In addition, the subcellular localization and kinase activities of PLK1 depend on the proper conformation of its polo-box domain [66, 67], and this conformation might also be maintained by HSP70. In addition to the importance of the SBD and ATPase domain, HSP70-CTD, in which the C-terminal CHIP/HOP-interacting IEEVD motif is deleted, also exhibited no rescue effects. Since we previously reported that this mutant hardly interacts with PCM components [31], this observation suggests a requirement for HSP70 interactions with the co-chaperone such as CHIP, which is particularly important in the chaperone-mediated proteasomal degradation, or HOP, which mediates the cooperation between HSP70 and HSP90 to achieve substrate folding [32, 65, 68, 69]. Since numerous PCM components undergo rapid turnover at the mitotic centrosome [12, 20, 63] and proteasomal activities were reported to control the centrosome protein level [30, 70, 71], and HSP90 has been reported to be a core member of Drosophila centrosome that regulates polo kinase stability and the function of mitotic centrosome [72, 73], we propose that HSP70-CHIP-mediated proteosomal degradation or HSP70-HOP-HSP90-mediated substrate folding may control the turnover of certain PCM components to facilitate mitotic PCM assembly. Notably, the alternate binding of HSP70 IEEVD motif to CHIP or HOP, and therefore the alternate substrate processing, is decided by phosphorylation near the C-terminal region of HSP70 [32], which is very close to the reported PLK1 phosphorylation site [33]. It would be interesting to investigate whether PLK1 phosphorylation of HSP70 modulate its interaction with the co-chaperones to achieve alternate substrate processing in the mitotic centrosome. Future identification of the HSP70 substrate proteins in the mitotic centrosome will be important to test this hypothesis.

## Conclusions

In conclusion, we have extended our previous finding that HSP70 is required for the functions of the mitotic centrosome in MT nucleation, bipolar spindle assembly and mitotic progression by showing that HSP70 acts through it chaperone activity to facilitate proper 3D assembly of PCM components at the mitotic centrosome. Since HSP70 activities and centrosome aberrations are well correlated with pathologies of cancer, age-related diseases, degeneration, and developmental defects, our findings may have broad-ranging applications in these conditions and provide information for future development of therapeutic strategies based on HSP70 or centrosome functions.

## Methods

### Cell culture and drug treatments

CGL2 and HeLa-S3 cells were obtained and cultured as previously described [31]. HeLa-S3 cells stably expressing EYFP-fused α-tubulin (HeLa-S3-tub-EYFP) were established by transfecting cells with pEYFP-tubulin vector (Clontech, Mountain View, CA, USA) and selecting stable transfectants in medium containing 1 mg/ml G418. Cells were routinely maintained in Dulbecco’s Modified Eagle’s Medium (Invitrogen, Carlsbad, CA, USA) supplemented with 10% fetal bovine serum (Invitrogen), 0.37% sodium bicarbonate, 100 U/ml of penicillin, and 100 μg/ml of streptomycin at 37 °C in an humidified incubator with 10% CO_2_; cells were passaged twice a week. PLK inhibitor III (Merck, Darmstadt, Germany), BI2536 (Selleckchem, Houston, TX, USA) and Pifithrin-μ (PES, Tocris Bioscience, Bistro, UK), were dissolved in dimethyl sulfoxide and stored at − 20 °C in aliquots. Treatment concentrations are given in Figure Legends, and unless otherwise indicated, all drugs were treated 30 min prior to cell fixation in order to examine immediate effects on the mitotic cells.

### Depletion and overexpression of HSP70

The shRNAs targeting *HSPA1A* (TRCN8757 and TRCN342860) and *HSPA1B* (TRCN8760 and TRCN 8763) were obtained from the National RNAi Core Facility Platform (Genomic Research Center, Academia Sinica). HSP70 in CGL2 or HeLa-S3-tub-EYFP cells were depleted as described [31]. The pFB-Neo vector containing FLAG-tagged wild-type (WT) HSPA1A was prepared as previously described [31]. Mutations of Lys^71^ to Glu, Glu^175^ to Ser, Ala^403^ to Gly, Val^438^ to Gly, and Val^438^ to Tyr were generated by site-directed mutagenesis. WT or mutant HSPA1A were ectopically overexpressed in cells as previously described [31]. For rescue experiments, cells stably overexpressing WT or mutant FLAG-HSPA1As were established under 1 mg/ml G418 selection and then transduced with shRNA targeting the 5′-untranslated region of *HSPA1A* (TRCN342860) to transiently deplete endogenous HSP70. Cells were also transduced with empty-vector pLKO.1 and pFB-Neo to provide controls for depletion and overexpression of HSP70, respectively.

### Immunofluorescence staining

Cells were seeded onto coverslips, incubated for 20 h before drug treatment or viral transduction, and then fixed in PTEMF buffer containing 20 mM PIPES, 4% paraformaldehyde, 0.2% Triton-X, 10 mM EGTA and 1 mM MgCl_2_ for 15 min. Primary antibodies included anti-HSP70 (GeneTex, Hsinchu, Taiwan or StressMarq, Victoria, Canada), anti-α-tubulin (GTX112141, GeneTex or T5168, Sigma, St. Louis, MO, USA), anti-γ-tubulin (T6557 or T3559, Sigma), anti-EB1 (E3406, Sigma), anti-pericentrin (ab4448 or ab28144, Abcam, Cambridge, UK), anti-CEP215 (IHC-00063, Bethyl, Cambridge, UK), anti-CEP164 (HPA037606, Sigma), anti-CEP152 (ab183911, Abcam), anti-PLK1 (No. 33-1700, Invitrogen), and anti-phospho-T210-PLK1 (PA1-126, ThermoFisher, Waltham, MA, USA). Alexa-Fluor 488-, 568-, 633-, or 647-conjugated goat anti-mouse or anti-rabbit IgG were purchased from Invitrogen, and Atto-488 goat anti-mouse IgG was from Sigma. Samples were mounted in Fluoromount-G from SouthernBiotech (Birmingham, AL, USA) for confocal or structured illumination microscopy (SIM) imaging. For ground state depletion (GSD) imaging, samples were mounted in DPBS (Sigma) containing 5% glucose, 100 mM 2-mercaptoethylamine (Sigma), 0.8 mg/ml glucose oxidase and 40 μg/ml catalase, and sealed with Twinsil (Picodent, #13001000, Wipperfürth, Germany).

### Confocal microscopic image acquisition and analysis

Confocal images of the stained cells were obtained from a Leica TCS-SP5 microscope with a HCX PLAPO 63×/1.4 objective. Images were acquired with 300 Hz scanning speed in 15 μm image stacks with a 0.5 μm step size. For the time-lapse images of mitosis progression, HeLa-S3-tub-EYFP cells were seeded in chambered coverslips (μ-slide 4 well, ibidi GmbH, Germany) and imaged on a Leica TSC-SP5 inverted microscope with a HCX PLAPO 40×/0.85 objective, 30 μm image stack, 0.5 μm step size and 10 min imaging span under a 37 °C and 10% CO_2_ environment. Laser intensity and HyD gain were fixed for each independent experiment, and the 3D confocal images were processed and projected into 2D with ImageJ. Quantitative analyses of confocal images were conducted with Imaris software. To determine the accumulation of HSP70 and other centrosome markers, a “Spot” of 1 or 2 μm in diameter surrounding the centrosome/spindle poles was created, and the intensity sum within the spot was measured. To determine the size of the centrosome, a “surface” covering the fluorescence signal of a centrosome marker was created, and the signal volume was measured. The conditions for immunostaining, imaging, and spot/surface creation were consistent for each independent experiment. Values for intensity and volume were normalized to markers that were stained and imaged in each experiment.

### 3D-SIM image acquisition and analysis

3D-SIM imaging was performed on a Zeiss ELYRA PS.1 LSM780 system with Plan-APOCHROMAT 63×/1.4 objective. A 28 μm grating for the 488-nm channel and 34 μm grating for the 568-nm channel with five rotations and five phases per z-section were used to image a stack covering the spindle pole region with 0.11 μm intervals in z-direction. Channels were aligned on ZEN software according to the reference landmark of multispectral beads (100 nm in diameter, Life technology). Percentage of colocalization between HSP70 and centrosome markers was then measured on Imaris software. To assess the relative spatial distribution of HSP70 and other centrosome markers from the SIM images, 3D image alignment and average were utilized on the Chimera software from UCSF [11, 41]. Briefly, all centrosome markers were co-stained with γ-tubulin, which served as a reference during the alignment process. The initial “top view” of γ-tubulin images were selected as the reference, as judged by the patterns of both γ-tubulin and the co-stained markers (e.g., CEP164). Other γ-tubulin images were then aligned to the reference to preserve the spatial relationship with the co-stained marker (CEP164). Once all the images were aligned, the γ-tubulin and CEP164 images were averaged separately to generate respective model images. Iterations of this process were then performed for other centrosome marker/γ-tubulin pairs to obtain the final 6-channel model images displaying relative distribution of all the centrosome markers. Images of HSP70, which was co-stained with γ-tubulin, CEP215 and PCNT, were then added by aligning the three PCMs to the model image. These 3D model images were then maxima-projected and channels were merged on ImageJ software.

### GSD image acquisition and analysis

GSD images were acquired from Leica SR GSD superresolution system with a HCX APO 100×/1.47 objective. Laser power was gradually increased until the repetitive photoswitching (blinking) of the single fluorophore molecule was observed; then the power was fixed, and the threshold was set to collect serial images of the fluorescence blinking. All single-molecule blinking events within ~ 10,000 serial images were localized and reconstructed into a high-resolution centrosome image. Laser power, threshold, and exposure time were kept constant for each independent experiment and all image acquisitions started from the 647-nm channel and proceeded to the 488-nm channel. To compare the spatial distribution and accumulation of PCNT and CEP215 at the mitotic centrosomes in control and HSP70-depleted cells, five lines intersecting at the center of PCNT GSD image were drawn and applied to the CEP215 channel for each GSD image, and the intensity along each line was measured, normalized, and averaged with ImageJ to obtain the mean distribution curve. For each line, the maximum intensity (peak) values within the 600 nm regions flanking the center were identified and the distance between two peaks was determined to verify the diameter of PCNT and CEP215 toroid. The maximum intensity (peak) values were also divided by the values at the center to obtain the peak-center intensity ratio to verify the accumulation of PCNT and CEP215. PLK1 recruitment to the spindle pole was measured with ImageJ, according to its intensity in the GSD images. A circle with a 1.2 μm diameter surrounding the spindle pole region was drawn. Normalized PLK1 intensity was then determined as the total intensity inside the circle divided by the total intensity outside the circle.

### Western blotting

Cell lysis and immunoblotting were carried out as described [31]. Specific proteins were detected using antibodies against HSP70 (GeneTex), GRP78 (610979, BD Biosciences, San Jose, CA, USA), HSC70 (sc-7298, Santa Cruz Biotechnology, CA), CEP215 (Bethyl), γ-tubulin (T6557, Sigma), EB1 (E3406, Sigma), GM130 (610822, BD Biosciences), FLAG (F3165, Sigma), respectively, and GAPDH (GTX100118, GeneTex) for use as loading controls.

### Proximity ligation assay (PLA)

PLA was used to test whether HSP70 regulates the interaction between PCNT and CEP215. Cells with or without HSP70 depletion were fixed and subjected for PLA with Duolink red starter kits (DUO92101, Sigma) as described previously [43]. Afterward, cells were mounted in Fluoromount-G containing DAPI and imaged with a Leica TCS-SP5 microscope. The PLA signal intensity was obtained by Imaris following the procedures described above.

## Data Availability

All data generated or analyzed during this study are included in the article.

## Abbreviations

GSD: ground state depletion
HSP70: heat shock protein 70
MT: microtubule
MTOC: MT organizing center
PCNT: pericentrin
PCM: pericentriolar material
PLK1: Polo like kinase 1
SIM: structured illumination microscopy
